# Acute trazodone administration reduces behavioral and physiological indicators of travel-induced anxiety in dogs in a road travel model

**DOI:** 10.3389/fvets.2026.1851849

**Published:** 2026-06-22

**Authors:** Gary Landsberg, Jessica Mendes, Whitney Kosziwka, Deborah A. Keys, Andria Gordon, Andrew King, Joseph A. Araujo, Christina de Rivera

**Affiliations:** 1 CanCog Inc., Toronto, ON, Canada; 2 Transpharmation Canada Ltd., Fergus, ON, Canada; 3 Kaleidoscope Statistics LLC, Athens, GA, United States; 4 Independent Consultant, Chatham, ON, Canada

**Keywords:** anxiety, behavior, canine, dog, gabapentin, road travel, stress, trazodone

## Abstract

**Introduction:**

Travel-induced stress and anxiety are prevalent in dogs resulting in behavioral changes that can negatively affect the overall well-being of dogs and their human travel companions. Although trazodone and gabapentin are not approved for use in dogs, they are commonly prescribed off-label for the management of situational fear and anxiety and both have demonstrated efficacy in alleviating stress during veterinary visits and noise fear. This study aimed to assess whether a single dose of gabapentin or trazodone could reduce or alleviate the physiological and behavioral signs of stress experienced by laboratory Beagles during road travel.

**Methods:**

Thirty-six dogs were selected for the study based on their baseline serum cortisol response to a single 10-min travel test in a van and were allocated to one of three groups (*N* = 12 dogs/group) that were balanced for cortisol response. Groups were then randomly assigned to one of three treatment groups: 10 mg/kg trazodone (9.4–12.8 mg/kg); 30 mg/kg gabapentin (27–35.7 mg/kg); or placebo control. The treatments were evaluated using a blinded, placebo-controlled, parallel group study design following oral administration at a single time point, 2 h prior to travel testing. During both baseline and treatment travel tests, serum cortisol and heart rate were assessed, as were behavioral measures with a primary focus on lip licking.

**Results:**

Administration of trazodone significantly reduced serum cortisol, lip licking frequency, lip licking scores, global anxiety scores, urination, yawning, and restlessness. However, mean heart rate was significantly higher following trazodone treatment compared to gabapentin both prior to and during the travel test and compared to control prior to the travel test. No differences were observed following gabapentin treatment relative to control on any of the physiological or behavioral assessment measures.

**Discussion/conclusion:**

These results support stress alleviating effects of trazodone for situational use, while demonstrating further support for the use of the travel test model for assessing the effects of therapeutic interventions on both physiological and behavioral signs of travel stress.

## 1 Introduction

Travel can be a stressful experience for many pet dogs, resulting in a range of behavioral and physiological responses that can negatively affect the well-being of both the dog and their owners and harm the human–animal bond. It is reported that 24–28% of dogs respond negatively to car transportation (1, 8). In addition to the stress experienced by the pet, car ride anxiety can result in significant stress for owners, including concerns about their pet’s welfare, and disruptive behaviors that are unsettling to passengers, and distracting to drivers which can pose a potential safety risk to human and pet passengers in the vehicle. Consequently, some owners may choose to limit or avoid traveling with their dogs, thereby restricting access to social outings, recreational activities, and other enrichment experiences for the pet and owner. Furthermore, travel anxiety may negatively impact veterinary care by delaying or reducing the frequency of visits and compromising the accuracy of vital signs and laboratory tests that may be altered by stress.

Stress is characterized by both physiological and behavioral responses resulting from the activation of the hypothalamic–pituitary–adrenal (HPA) axis and the subsequent release of glucocorticoids and catecholamines. Studies investigating travel-related stress in dogs have demonstrated significant increases in cortisol levels, heart rate, and neutrophil-lymphocyte ratios, along with significant decreases in heart rate variability and increased expression of behavioral signs associated with fear, anxiety, and frustration (2–7). Commonly observed behaviors in these studies include lip licking, panting, restlessness, escape attempts (climbing and digging), yawning, vocalization, and salivation (3–6, 40), all of which are among the most frequently owner-reported signs in dogs that respond negatively to travel (1, 8). Evidence from multiple studies indicates that increases in cortisol, heart rate, and lip licking are generally the most consistent and reliable indicators of travel-induced stress across repeated exposures (3–7, 9, 10) and were therefore selected as the primary outcome measures in the current study. Collectively, these behaviors are widely recognized indicators of fear and anxiety-related responses in dogs and have been shown to be reliably elicited and quantified across repeated travel exposures, supporting the behavioral validity of controlled road-travel models for the assessment of anxiety-reducing interventions.

Treatment to alleviate travel anxiety focuses on making travel safer and more comfortable for both dogs and their owners. Behavior counseling includes management strategies to avoid travel that might have unpleasant consequences for the pet’s emotional well-being and to prevent further negative associations with travel, together with positive reinforcement-based behavior modification to desensitize and counter-condition dogs to travel. While these treatments can be highly effective with time and compliance, pharmaceutical interventions for situational use are often needed to address the immediate welfare needs of pets and owners when travel is necessary or unavoidable, such as for veterinary visits, and as an adjunct to the behavior modification program. Furthermore, anxiolytic interventions have been demonstrated to significantly improve both behavioral and physiological signs associated with travel stress (3, 4, 6, 9–11).

Trazodone and gabapentin are two of the most recommended and prescribed drugs for managing fear, anxiety, and stress related to travel and veterinary visits in dogs (12–16, 45). Both demonstrate anxiolytic efficacy in humans (17–20). As neither is approved for use in dogs, the human versions are prescribed off-label. In primary care practice, trazodone has been reported to be prescribed to 8.4% of dogs with categorized behavioral problems and 1.33% of all patients, which is 28x higher than clomipramine and fluoxetine combined (21). Its use has increased dramatically since 2015, reflecting more recent research on its use in dogs in stressful situations (22, 23) and corresponding to the launch of the Fear Free™ program, which emphasizes the value of psychotropic drugs for fearful and anxious pets, particularly for travel and veterinary visits (21). Similarly, in a recent study from North American shelters (*N* = 129) trazodone (69%) and gabapentin (61%) were the most commonly used psychoactive medications in dogs with trazodone having a slightly higher efficacy score (24).

Trazodone is a potent antagonist of serotonin 5-HT_2A_ and 5-HT_2C_ receptors, a partial agonist of 5-HT_1A_ receptors and a serotonin reuptake inhibitor (18, 25). It has been shown to be effective in reducing stress during veterinary visits and for noise fears at doses of 5–12 mg/kg (14, 26) and to facilitate postoperative confinement (22). Gabapentin, while structurally similar to gamma-aminobutyric acid (GABA), does not bind to GABA receptors and does not affect GABA uptake or metabolism. Although its exact mechanism of action as an anxiolytic is not fully understood, gabapentin binds to the alpha-2-delta subunits of voltage-gated calcium channels, which may reduce calcium influx and inhibit the release of excitatory neurotransmitters, such as glutamate and norepinephrine (19, 25, 27, 28). Gabapentin has been shown to be effective in reducing veterinary stress and storm phobia in dogs at doses of 25–50 mg/kg (16, 29).

This study aimed to assess whether a single dose of gabapentin or trazodone would reduce or alleviate the physiological and behavioral signs of stress in a laboratory model of road travel stress and anxiety. The use of a standardized travel model, including a controlled and consistent environment (e.g., transport cage and vehicle), fixed routes and durations, and continuous video recording and monitoring equipment, combined with physiological and behavioral analyses conducted by trained technicians, provides a practical, uniform, and rigorous approach to evaluate stress responses and treatment efficacy (3–7, 9–11, 40).

## 2 Materials and methods

### 2.1 Subjects and husbandry

Forty-four Beagle dogs, maintained in the Transpharmation Canada Ltd. colony for a minimum of 1 month prior to study initiation, entered the study at baseline; thirty-six dogs were selected to enter the treatment phase. The subjects were in good general health and were either naïve to travel testing or previously had shown serum cortisol elevation in response to travel (i.e., over 90 nmol/L). The selected dogs included 16 neutered males and 20 spayed females between 1.9 and 9.6 years of age (mean age 5.1 years). Baseline group demographics are included in Table 1.

**Table 1:** Baseline group demographics.

Animal ID	Sex	Date of birth (YYYY-MM-DD)	Age (years)	Baseline pre-travel cortisol (nmol/L)	Baseline post-travel cortisol (nmol/L)	Testing cohort	Body weight (kg)	Treatment assignment
1	M	2015–02–17	9.3	59	188	1	13.8	Trazodone
2	F	2016-09-01	7.7	37	151	1	9.8	Trazodone
3	F	2021-07-30	2.8	48	323	1	9.0	Trazodone
4	F	2016-09-16	7.7	50	262	1	10.4	Trazodone
5	M	2015–02–16	9.3	35	246	2	9.6	Trazodone
6	F	2015–02–16	9.3	31	199	2	11.9	Trazodone
7	F	2014–10-27	9.6	29	183	2	11.4	Trazodone
8	M	2020-01-16	4.3	60	227	2	12.8	Trazodone
9	M	2021-03-14	3.2	55	244	2	9.7	Trazodone
10	F	2018–11–24	5.5	43	231	2	10.6	Trazodone
11	F	2021-07-19	2.8	20	201	2	9.4	Trazodone
12	F	2021-07-09	2.8	41	270	2	7.8	Trazodone
Mean				42.33	227.08		10.52	
13	M	2020–01–23	4.3	116	206	1	15.4	Gabapentin
14	F	2018–07–17	5.8	77	261	1	10.7	Gabapentin
15	M	2022-06-17	1.9	57	245	1	10.9	Gabapentin
16	M	2015–02–14	9.3	35	312	1	11.3	Gabapentin
17	F	2021-07-28	2.8	55	272	1	8.4	Gabapentin
18	F	2018-04-05	6.1	42	171	1	11.1	Gabapentin
19	M	2021-07-30	2.8	40	192	1	9.2	Gabapentin
20	M	2015–02–15	9.3	51	154	1	12.0	Gabapentin
21	M	2022-06-09	1.9	42	239	2	9.8	Gabapentin
22	F	2021-07-07	2.8	38	221	2	7.0	Gabapentin
23	F	2021-07-27	2.8	42	198	2	8.9	Gabapentin
24	M	2022-06-17	1.9	22	252	2	10.2	Gabapentin
Mean				51.42	226.92		10.41	
25	F	2017-12-07	6.4	40	275	1	9.2	Control
26	M	2021-07-30	2.8	81	242	1	10.4	Control
27	F	2021-07-07	2.8	65	194	1	7.2	Control
28	M	2022-06-30	1.9	56	275	1	9.6	Control
29	F	2018-09-30	5.7	41	255	1	10.7	Control
30	F	2016-08-28	7.8	31	197	2	11.9	Control
31	F	2015–02–15	9.3	63	220	2	10.3	Control
32	M	2020-04-30	4.1	26	164	2	12.2	Control
33	M	2022-06-09	1.9	38	217	2	11.0	Control
34	M	2015–02–17	9.3	25	160	2	11.0	Control
35	F	2021-07-18	2.8	36	241	2	8.1	Control
36	F	2021-07-23	2.8	40	250	2	8.4	Control
Mean				45.17	224.17		10.00	

Dogs were housed in groups of up to four in pens measuring 1.52 × 4.9 m, which included raised resting platforms and a rotation of toys for enrichment. During the treatment phase, the dogs were housed according to their treatment and cohort assignment, such that dogs from the same treatment group and testing cohort were housed together. Dogs were individually fed to maintain their body condition using a commercially available diet: Purina® ProPlan® All Ages Sport Active 27/17 Chicken and Rice Formula. Dogs were fed once daily at the end of the day following the completion of all study-related procedures. Water was not offered during travel assessments but was otherwise provided *ad libitum*.

### 2.2 Study design

Prior to the initiation of baseline procedures, the dogs were listed in alphabetical order. The first 22 dogs were assigned to cohort 1 and the remaining 22 dogs were assigned to cohort 2. The dogs remained in their assigned cohort for the duration of the study to ensure that the time between testing for each subject was consistent (i.e., 5 days between baseline and treatment phase assessment). The procedures for each cohort were separated by 1 day. During the baseline assessment and screening phase, dogs underwent a single 10-min travel test in a cargo van which included activity and behavioral assessments, evaluation of body position, continuous heart rate monitoring, and analysis of both pre- and post-test serum cortisol levels.

Following baseline assessments, dogs were ranked according to their post-travel serum cortisol levels, such that the dog with the highest cortisol value received a rank of 1. Any dog with the same cortisol value was ranked alphabetically by given name. The six dogs with the lowest levels of post-travel serum cortisol, one dog that reached a predetermined humane endpoint of two vomiting events during travel, and one dog with a vomiting event during travel that also had a previous history of vomiting during travel were excluded from the study.

The remaining 36 dogs showed baseline post-travel cortisol responses that exceeded the minimum level that we have established as a stress response in our research colony (over 90 nmol/L) were then allocated to one of three treatment groups (*N* = 12 dogs/group) balanced for cortisol response and body weight to the extent possible, using a 1-2-3-3-2-1 block pattern such that ranks 1, 2, and 3 were assigned to groups 1, 2, and 3, respectively, and ranks 4, 5, and 6 were assigned to groups 3, 2, and 1, respectively. This pattern continued until all dogs were placed in a group. The groups were then adjusted slightly to ensure balance for previous exposure to travel within 1 month of study initiation (i.e., two dogs per group). Group exchanges were performed between dogs with similar cortisol responses, and the finalized treatment groups did not differ significantly in baseline body weight. The groups were then randomly assigned to one of the following treatment conditions using a random number generator in Microsoft Excel® by a designated team of unblinded technical staff: (1) 10 mg/kg trazodone; (2) 30 mg/kg gabapentin; or (3) placebo control. To ensure groups were balanced at baseline following allocation, independent *t*-tests were conducted comparing each post-travel serum cortisol among all group combinations.

Sample size was determined prospectively based on effect sizes observed in prior travel studies using similar physiological and behavioral outcome measures, with serum cortisol designated as the primary physiologic endpoint for sample size considerations. Significant reductions in serum cortisol, as well as heart rate and lip-licking behavior were previously observed following treatment with dexmedetomidine oromucosal gel in a preliminary crossover design study with 12 dogs using this study model (10). A significant reduction in cortisol has also been demonstrated following a single dose of CBD in a placebo-controlled design in a similar car travel study of 19 dogs (ten control and nine treatment) (6). In addition, an unpublished, internal *a priori* power analysis for cortisol as the primary endpoint, using this same road travel model, determined that a sample size of 12 dogs would be sufficient to detect a 15% difference at a significance level of 0.05 and a power of 0.8. Based on these considerations, the present study was designed to be adequately powered for detection of treatment effects on cortisol as the primary endpoint, with lip-licking designated as the principal behavioral endpoint.

In a blinded, parallel group design, the assigned treatments were orally administered at a single time point on the day of testing, 2 h prior to travel. Treatment phase assessments were performed 5 days after the baseline assessment for each dog. Activity and behavioral assessments, evaluation of body position, continuous heart rate monitoring, and analysis of both pre- and post-test serum cortisol levels were performed as described at baseline. General health observations were performed twice daily during the study period. See Table 2 for the study schedule.

**Table 2:** Study schedule.

Study day^a^		Phase	Procedures
Cohort 1	Cohort 2		
−5	−5	Baseline	Beginning of twice daily health observations
−5	−4		Pre-test blood sampling for cortisol analysis (at least 3 h pre-test)
			Continuous heart rate monitoring (pre-test and for duration of test)
			10-min travel test
			Behavioral analysis (live during test and from saved video files)
			Post-test blood sampling for cortisol analysis (within 8 min of test end)
−3	−3		Body weight measurements
−2	−2		Group allocation
			Rehousing (by treatment group and cohort)
0	1	Treatment	Pre-test blood sampling for cortisol analysis (at least 3 h pre-test)
			Test/control article administration (2 h prior to test)
			Continuous heart rate monitoring (pre-test and for duration of test)
			10-min travel test
			Behavioral analysis (live during test and from saved video files)
			Post-test blood sampling for cortisol analysis (within 8 min of test end)

a Study day—5 corresponded to 2024-05-08, and the treatment phase (study days 0–1) was completed by 2024-05-14.

### 2.3 Dosing

The assigned treatments of 10 mg/kg trazodone (9.4–12.8 mg/kg), 30 mg/kg gabapentin (27–35.7 mg/kg), or placebo control were orally administered at a single time point on the scheduled test day by trained technical staff, and all animals received their assigned intervention. Each dog was dosed 2 h (±19 min) prior to their travel assessment. Doses were calculated according to baseline body weight measurements, following the dosing guidelines outlined in Tables 3, 4.

**Table 3:** Gabapentin dosing guidelines.

Body weight (kg)	Dose (mg)
<7.5	200
7.5 to <11.7	300
11.7 to <13.4	400
≥13.4	500

Gabapentin capsules (Apotex Inc.) were available at doses of 100 mg and 300 mg. As capsule splitting was not practical, dose guidelines outlined in Table 3 were used. Trazodone tablets (Apotex Inc.) were available in 50 mg and 100 mg strengths. As the tablets were scored, it was practical to administer doses in 25 mg increments according to the dosing guidelines outlined in Table 4. The control product was a scored tablet, and the number of administered tablets was matched to that of the 10 mg/kg trazodone dose group. The dogs were individually housed and observed for 30 min following dosing for signs of vomiting or dose regurgitation; however, no such incident occurred.

**Table 4:** Trazodone dosing guidelines.

Body weight (kg)	Dose (mg)
<6.3	50
6.3 to <7.5	75
7.5 to <11.3	100
11.3 to <12.5	125
≥12.5	150

### 2.4 Travel assessments

**Figure 1:**
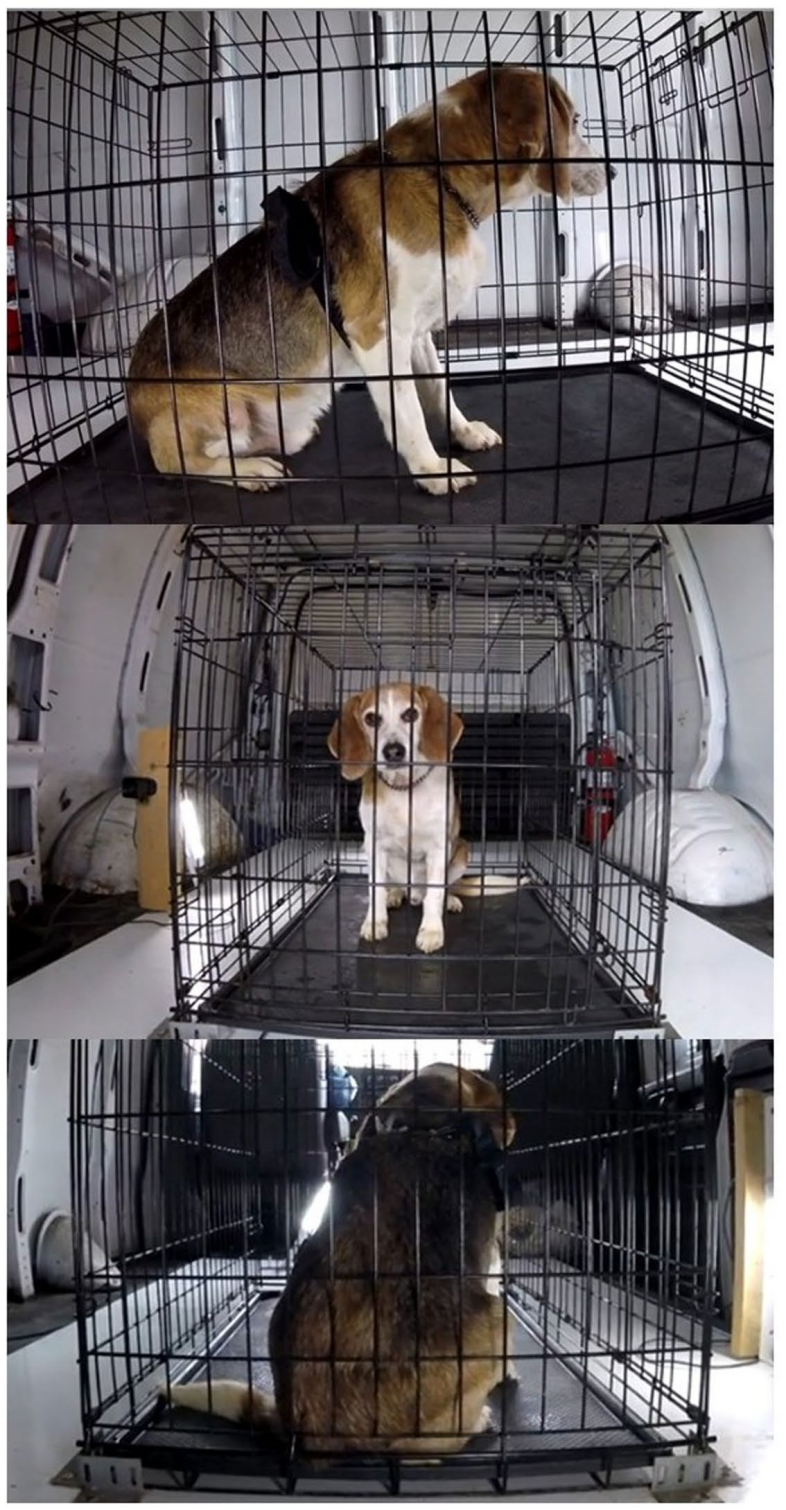
Travel test setup showing a dog in the crate being monitored from three camera angles while wearing the heart rate monitor.

Dogs were removed from their home pen approximately 10 min prior to their individual travel assessment and 3 min of pre-travel heart rate data were collected a minimum of 5 min prior to the start of travel (heart rate collection procedures are described below). The dog was then placed inside a crate (24 × 30 inches) secured in the back of a cargo van. The crate consisted of wire mesh sides and a plastic floor covered with a rubber mat to provide traction. Prior to each assessment, the crate and mat were cleaned to prevent odors from affecting test performance. Three video cameras were mounted within the vehicle to record the dog’s behavior from various angles (front, back, and one side view; see Figure 1). Two technicians were present in the van. One person operated the vehicle, and the second person monitored the animal for behavioral parameters, as described below. The test lasted 10 min and sessions were conducted in an identical manner for each dog to the extent possible. The travel route began and ended with an unpaved driveway and rural roadway, transitioning to paved roads of up to 80 km per hour, with one midway turnaround point. The driver attempted to keep the speed of each session consistent. The dogs were tested at approximately the same time of day for both baseline and treatment assessments (±53 min). All travel assessments were performed during daylight hours and the temperature within the vehicle was maintained within a range of approximately 15 °C to 25 °C.

### 2.5 Behavioral analysis during travel

The behavior of each dog was assessed by direct observation during travel by the passenger technician and from saved video files. Passenger observations included manual recording of vomiting, urination, and defecation events in 5-min intervals. Each behavior was scored as “1” for present, or “0” for absent. In addition, the frequency of vocalization was recorded in 2.5-min intervals and later converted to a score of 0–3 as outlined in Table 5. Video footage from the three cameras mounted within the van was merged into a single file and used to assess changes in body position (i.e., frequency and duration of standing, sitting, and lying down) and lip licking frequency in 2.5-min intervals. These behaviors were recorded via keypress in EventMark software (Transpharmation Canada Ltd.). Lip licking frequency was then converted to a score of 0–4 as outlined in Table 5. The scoring of yawning, panting, salivating, movement/turning, and escape were measured for each 2.5-min interval using manual count tallies for event or duration (see Table 5) by the behavioral analyst scoring the videos. A global anxiety score was then calculated by adding all scores outlined in Table 5 for each 2.5-min interval (maximum score 88; see Table 6). All behavioral assessments from video recordings were performed by a single registered veterinary technician (RVT) trained and experienced in conducting behavioral evaluations within our travel model to ensure consistency across all scoring sessions and intra-observer reliability was assessed.

**Table 5:** Scoring scale of behaviors included in the global anxiety score.

Scored for each 2.5-min interval					
Behavior	Score 0	Score 1 infrequent	Score 2 occasional	Score 3 frequent	Score 4 extensive
Vocalization	Not present	1–2 events	3–5 events	>5 events	
Lip licking	Not present	1–3 events	4–10 events	11–20 events	>20 events
Panting	Not present	1–2 events or <5 s	3–5 events or 5–30 s	>5 events or >30 s	
Salivating/drooling	Not present	Minimal/mildly damp	Moderate drooling	Intense/excessive pooling of saliva	
Escape attempts/frustration^a^	Not present	1–2 events or <5 s	3–5 events or 5–30 s	>5 events or >30 s	
Restlessness/repeated movement^b^	Not present	1–2 events or < 5 s	3–5 events or 5–30 s	>5 events or >30 s	
Yawn/mouth gape	Not present	1–2 events or <5 s	3–5 events or 5–30 s	>5 events or >30 s	

a Digging, scratching or pawing at crate sides, floor or door, or biting or climbing at the sides of door.

b Controlled lateral or circling movement of two legs, excluding stumbling or loss of balance due to vehicle movement.

**Table 6:** Mean ±SD scores of each behavior included in the global anxiety score.

Treatment	Trazodone		Gabapentin		Control	
Phase	Baseline	Treatment	Baseline	Treatment	Baseline	Treatment
Vocalization score	1.67 ± 3.26 (*n* = 3)	0.33 ± 0.65 (*n* = 3)	1.67 ± 3.26 (*n* = 4)	1.50 ± 2.71 (*n* = 4)	0.08 ± 0.29 (*n* = 1)	0.25 ± 0.87 (*n* = 1)
Lip licking score	8.00 ± 3.84 (*n* = 12)	2.17 ± 1.99 (*n* = 9)	9.58 ± 1.24 (*n* = 12)	7.50 ± 3.97 (*n* = 12)	7.92 ± 3.53 (*n* = 12)	7.75 ± 3.55 (*n* = 12)
Panting score	1.00 ± 2.37 (*n* = 2)	0.00 ± 0.00 (*n* = 0)	1.33 ± 1.97 (*n* = 5)	0.42 ± 1.00 (*n* = 2)	1.67 ± 3.14 (*n* = 3)	2.17 ± 4.06 (*n* = 4)
Salivating score	1.17 ± 2.59 (*n* = 4)	0.00 ± 0.00 (*n* = 0)	1.25 ± 1.66 (*n* = 5)	0.25 ± 0.62 (*n* = 2)	0.75 ± 1.36 (*n* = 3)	0.25 ± 0.62 (*n* = 2)
Escape score	0.50 ± 1.17 (*n* = 2)	0.17 ± 0.39 (*n* = 2)	0.58 ± 1.00 (*n* = 4)	0.83 ± 1.47 (*n* = 5)	0.25 ± 0.62 (*n* = 2)	0.33 ± 0.89 (*n* = 2)
Restlessness score	3.83 ± 2.72 (*n* = 12)	1.67 ± 1.50 (*n* = 9)	4.50 ± 2.97 (*n* = 12)	4.25 ± 3.08 (*n* = 11)	4.75 ± 3.02 (*n* = 12)	4.67 ± 2.87 (*n* = 11)
Yawning score	0.92 ± 1.08 (*n* = 6)	0.25 ± 0.62 (*n* = 2)	1.75 ± 1.22 (*n* = 10)	1.33 ± 0.98 (*n* = 10)	1.17 ± 1.19 (*n* = 7)	0.92 ± 0.90 (*n* = 8)
Global anxiety total score	17.08 ± 9.03	4.58 ± 3.34	20.67 ± 8.18	16.08 ± 7.40	16.58 ± 9.33	16.33 ± 8.92

*n* = number of individuals exhibiting the behavior out of 12 dogs per treatment group.

### 2.6 Heart rate measurement

Continuous heart rate measurements were taken in 1 s intervals, prior to and during each travel test using the Polar H10 Bluetooth heart rate sensor and associated chest strap. Prior to the commencement of the baseline phase, dogs that had not recently received acclimation to the test strap (or were completely naïve) were acclimated for sessions of ≥10 min in duration. Dogs previously exposed to the strap underwent a single acclimation session, and dogs not previously exposed underwent two sessions.

Before each travel test, the dog was removed from the housing pen and brought to an adjacent procedure room. The chest strap was placed on the animal just behind the forelimbs, and the sensor was positioned on the left side of the chest where a small patch of fur had been shaved a minimum of 1 day prior. Contact gel was used to improve the functionality of the sensor. Following placement, the dog was acclimated to the strap for a minimum of 2 min. This was followed by 3 min of heart rate recording while the animal was at rest. The dog was then placed into the travel crate within the vehicle, and heart rate data were collected for the entire duration of the test.

### 2.7 Serum cortisol analysis

Blood samples were collected for analysis of serum cortisol levels before and after each travel test. All samples were collected by direct venipuncture from a jugular vein. Pre-travel samples were collected en masse at the beginning of the day, at least 3 h prior to the test. Immediately following travel, the dog was removed from the vehicle and placed into an individual cage within the test facility until the time of blood collection, which was 8–10 min after the end of the test. Travel testing was scheduled so that post-travel blood collections were performed at approximately the same time of day for each dog (±53 min). At each time point, blood was collected into a serum separator tube and left to clot at room temperature for a minimum of 10 min prior to centrifugation at 2800–3200 rpm for 10 min at 20 °C. Tubes containing the isolated serum were refrigerated at 2–8 °C until they were sent on wet ice to Antech Diagnostics Canada Ltd. for analysis on the day of collection. Serum cortisol concentrations were quantified using a competitive homogeneous enzyme immunoassay.

### 2.8 Health observations and humane endpoint monitoring

The health of the dogs was observed twice daily over the course of the study, with AM and PM observations separated by a minimum of 6 h. The animals were observed for any signs of health or behavior that would not be expected in normal, healthy dogs. Humane endpoints specific to the road-travel assessments were predefined to ensure animal welfare during testing. Criteria for removal from travel or immediate intervention included a severe stress response during travel, defined as the occurrence of two or more of the following during a single travel test: uncontrolled defecation or urination, intense and persistent escape behaviors with potential risk of injury, or vomiting at a frequency of two events.

### 2.9 Endpoint classification

The primary endpoint for this study was serum cortisol. Lip licking was considered to be the principal behavioral endpoint, which was analyzed for both frequency and score (magnitude of response). Global anxiety, change in body position, and heart rate were secondary endpoints. As the other behavioral measures are less frequently, reliably, and/or consistently observed, they were not specified as endpoints in the study protocol. However, as restlessness, yawning and the presence or absence of urination were observed with sufficient frequency and distribution across treatment groups, these were analyzed post-hoc as exploratory measures.

### 2.10 Statistical analysis

Statistical analyses were performed using SAS 9.4 (Cary, NC) except for Dunn’s test and intraclass correlation coefficients (ICC) which were performed using the dunn.test (Version 1.3.5, 2017) and irr (Version 0.84.1, 2012) packages, respectively, in R (R Core Team). A significance threshold of *p* < 0.05 was used for all endpoints. All animals enrolled in the study were included in the statistical analysis.

To test experimental model validity, baseline pre-travel serum cortisol levels and heart rates were compared to baseline post-travel cortisol and during travel heart rates, respectively, using paired t-tests.

**Figure 2:**
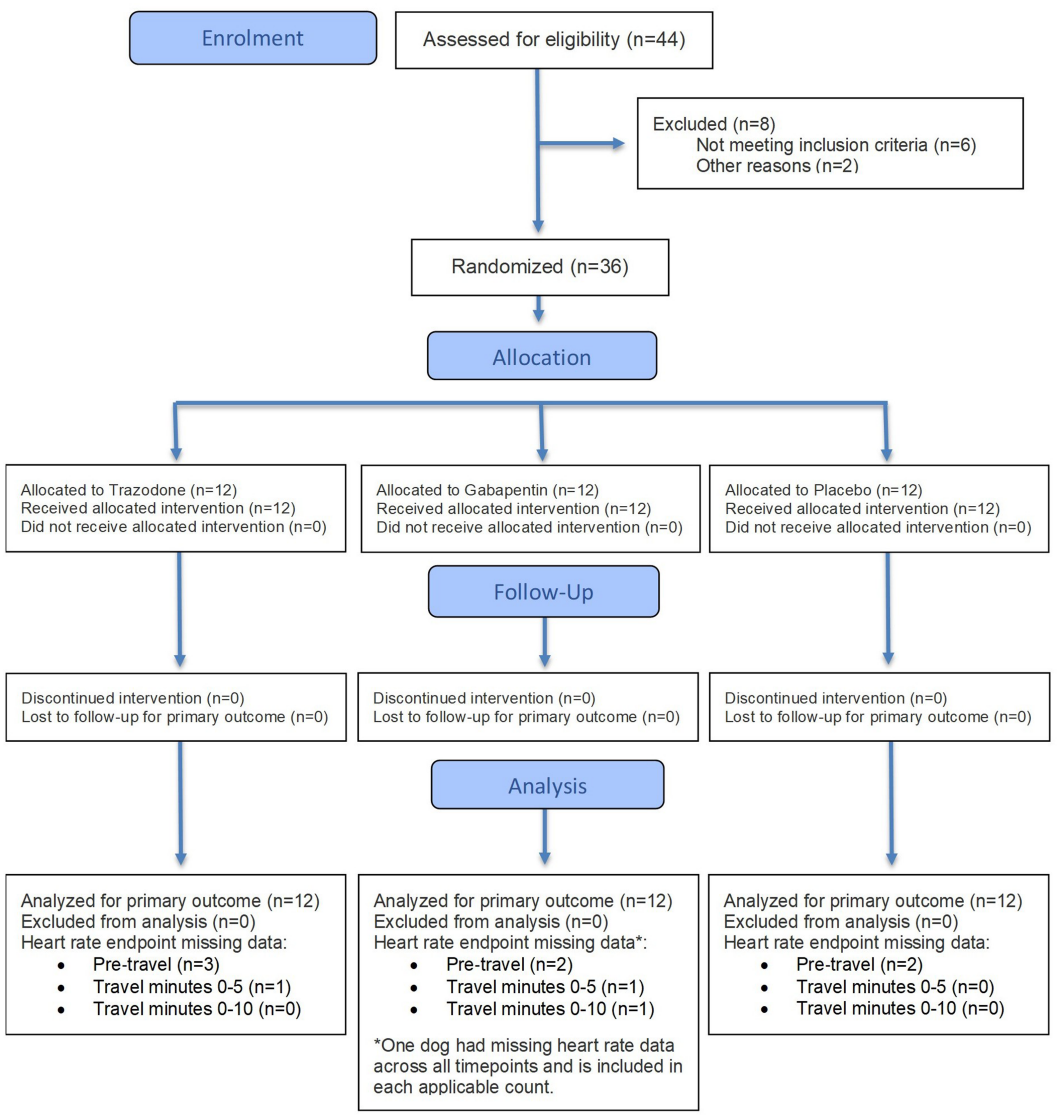
Flow diagram of animal selection, allocation, and analysis populations.

All outcomes were compared between treatment groups during treatment phase. Post-travel serum cortisol levels, heart rates measured both before and during travel, global anxiety scores, restlessness scores and converted lip licking scores were analyzed using linear models. For heart rate endpoints, occasional missing values occurred due to loss of sensor functionality/connectivity; analyses were performed using available observations for each endpoint and timepoint. This resulted in seven dogs being excluded from the pre-travel analysis (3 trazodone, 2 control, and 2 gabapentin), two dogs excluded from the first 5-min analysis (1 trazodone and 1 gabapentin), and one dog in the gabapentin group excluded from the full 10-min analysis, with the same dog from the gabapentin group accounting for the missing data across all analysis periods (see Figure 2). Lip-licking frequencies were analyzed using negative binomial regression as appropriate for count data. Presence of urination and yawning during travel was analyzed using logistic regression. Each model included treatment group, cohort, and baseline phase value as a covariate: post-travel baseline phase value for cortisol or during-travel baseline phase value for all other outcomes. Additionally, the model for cortisol included a pre-travel treatment phase serum cortisol covariate to control for initial variability in pre-travel values. Sex, age, and prior travel exposure were not included as covariates in the statistical models. Prior travel exposure within 1 month of study initiation was considered during subject selection and group allocation to support balance across treatment groups but was not included in the final statistical models. Normality of linear model residuals was confirmed by examining histograms and Q–Q plots. Quasi-separation was present in the urination analysis; therefore, Firth’s bias-reduced penalized-logistic regression was used. Kruskal–Wallis tests were used to compare lying, sitting, and standing durations during travel and their respective change from baseline phase durations between groups. Multiple comparisons between the three treatments were adjusted for with Tukey’s tests for linear and negative binomial regression models and with Dunn’s tests for Kruskal–Wallis tests.

Fisher’s exact tests were alternatively used to compare the proportion of dogs that had global anxiety scores, and lip licking frequencies that decreased from baseline between treatment groups. A Fisher’s exact test was also used to compare the proportion of dogs that had post-travel cortisol values beneath the established stress response threshold between treatment groups.

A two-way random effects model with single rater/measurement and absolute agreement was used to calculate ICCs for agreement between original and rescored behavioral scores. The interpretation of ICC was based on the guidelines proposed by Koo and Li (30), where ICC values >0.9 were considered excellent.

## 3 Results

A flow diagram of animal disposition and analysis populations are summarized in Figure 2.

### 3.1 Intra-observer reliability assessment

Intra-observer reliability for all behavioral measures was excellent, with ICCs equal to or exceeding 0.96 across all behavioral categories, confirming that the rescoring process closely replicated the original measurements.

### 3.2 Baseline physiological measures and model validity

**Figure 3:**
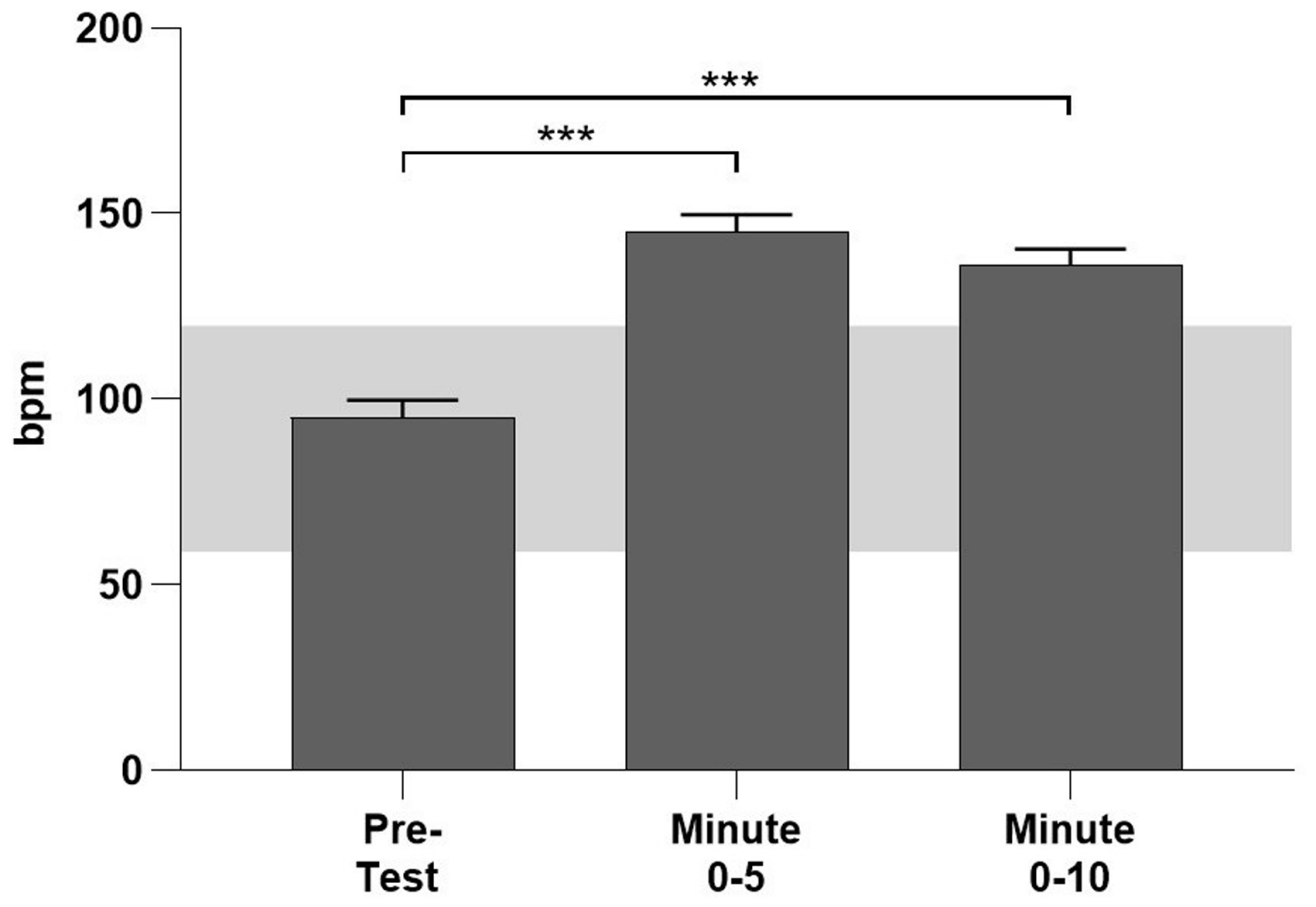
Mean + SEM baseline heart rate (bpm) prior to travel and during travel at minutes 0–5 and 0–10. Asterisks indicate significant differences between measurement periods (***p<0.0001). The shaded area represents a normal resting heart range of 60–120 bpm.

**Figure 4:**
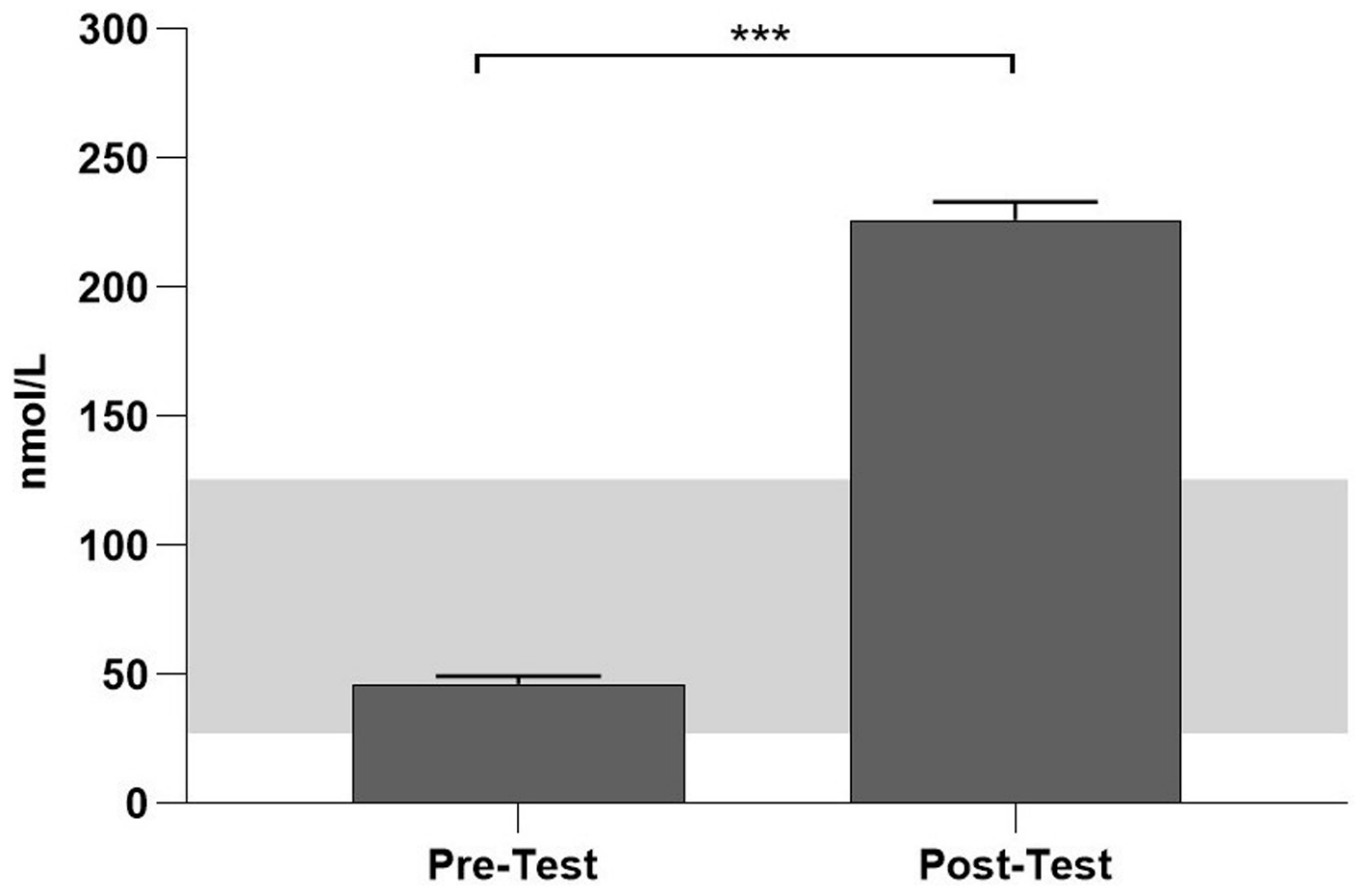
Mean + SEM baseline serum cortisol levels (nmo/L) prior to travel and 8 min post-travel. The asterisk indicates a significant difference between the time points (^***^p < 0.001). The shaded area represents the diagnostic laboratory’s normal cortisol reference range of 28–124 nmol/L.

Baseline assessments resulted in measurable and significant physiological stress responses to the travel tests. Heart rates were elevated for both the first 5 min and the entire duration of the travel test compared to heart rates pre-test. The mean heart rate during baseline travel increased by 40 bpm (95%CI 28–53) from the mean heart rate pre-travel (*p* < 0.001). In addition, the mean heart rate during travel minutes 0–5 increased by 49 bpm (95%CI 36–62) over the pre-travel mean heart rate (*p* < 0.001) (see Figure 3). The mean cortisol level at baseline was also elevated post-test and increased by 180 nmol/L (95%CI 165–195) compared to pre-test (*p* < 0.001) (see Figure 4). All 36 dogs selected for the study showed baseline post-travel cortisol responses that exceeded our research colony’s established stress response threshold of 90 nmol/L as well as the upper limit of the diagnostic laboratory’s reference range (124 nmol/L). Therefore, all dogs included in this study exhibited significant physiological stress responses in cortisol and heart rate at baseline for the evaluation of the efficacy of the test products during the treatment phase. In addition to these physiological responses, during baseline travel, dogs exhibited behavioral signs associated with fear and anxiety, including lip licking, yawning, and restlessness, supporting the validity of these behavioral measures for evaluating treatment effects.

### 3.3 Serum cortisol

**Figure 5:**
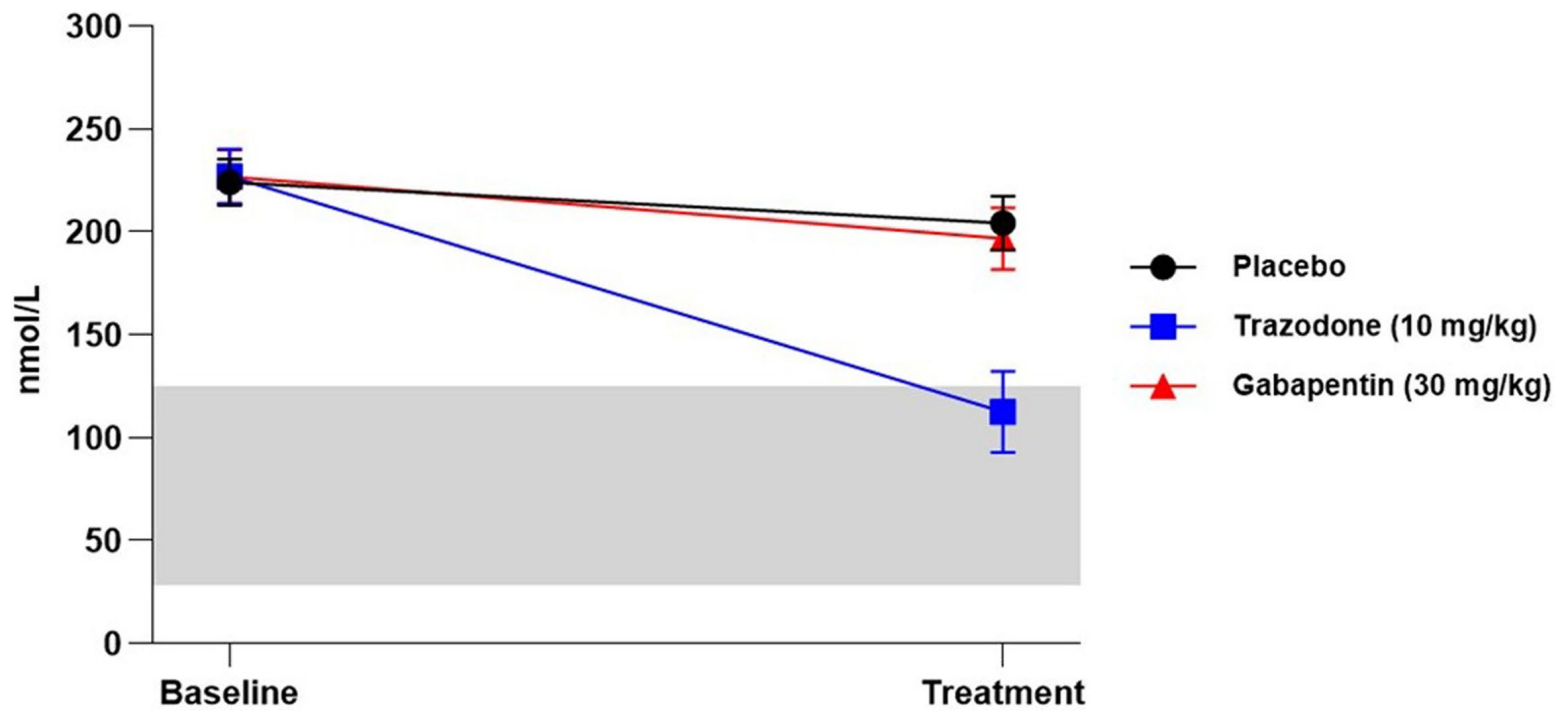
Mean ± SEM serum cortisol levels (nmol/L) measured 8 min post-travel by treatment type and study phase. Adjusted post-travel serum cortisol levels were significantly lower in the trazodone-treated group than in the gabapentin (*p* = 0.02) and control groups (*p* < 0.001). The shaded area represents the diagnostic laboratory’s normal cortisol reference range of 28–124 nmol/L.

The adjusted mean post-travel serum cortisol level was significantly lower in the trazodone-treated group than the gabapentin group by 89 nmol/L (95%CI 31–146) (*p* = 0.02) and the control group by 94 nmol/L (95%CI 38–149) (*p* < 0.001, Figure 5). There were also significant group differences in the number of dogs with post-travel cortisol values below the established stress response threshold of 90 nmol/L with 42% (5/12) of dogs in the trazodone-treated group, 8% (1/12) in the gabapentin group, and 0% in the control group (*p* = 0.03). In addition, for two of the 12 dogs in the trazodone group, cortisol levels did not increase post-travel while they increased for the remaining 34 dogs.

### 3.4 Heart rate

**Figure 6:**
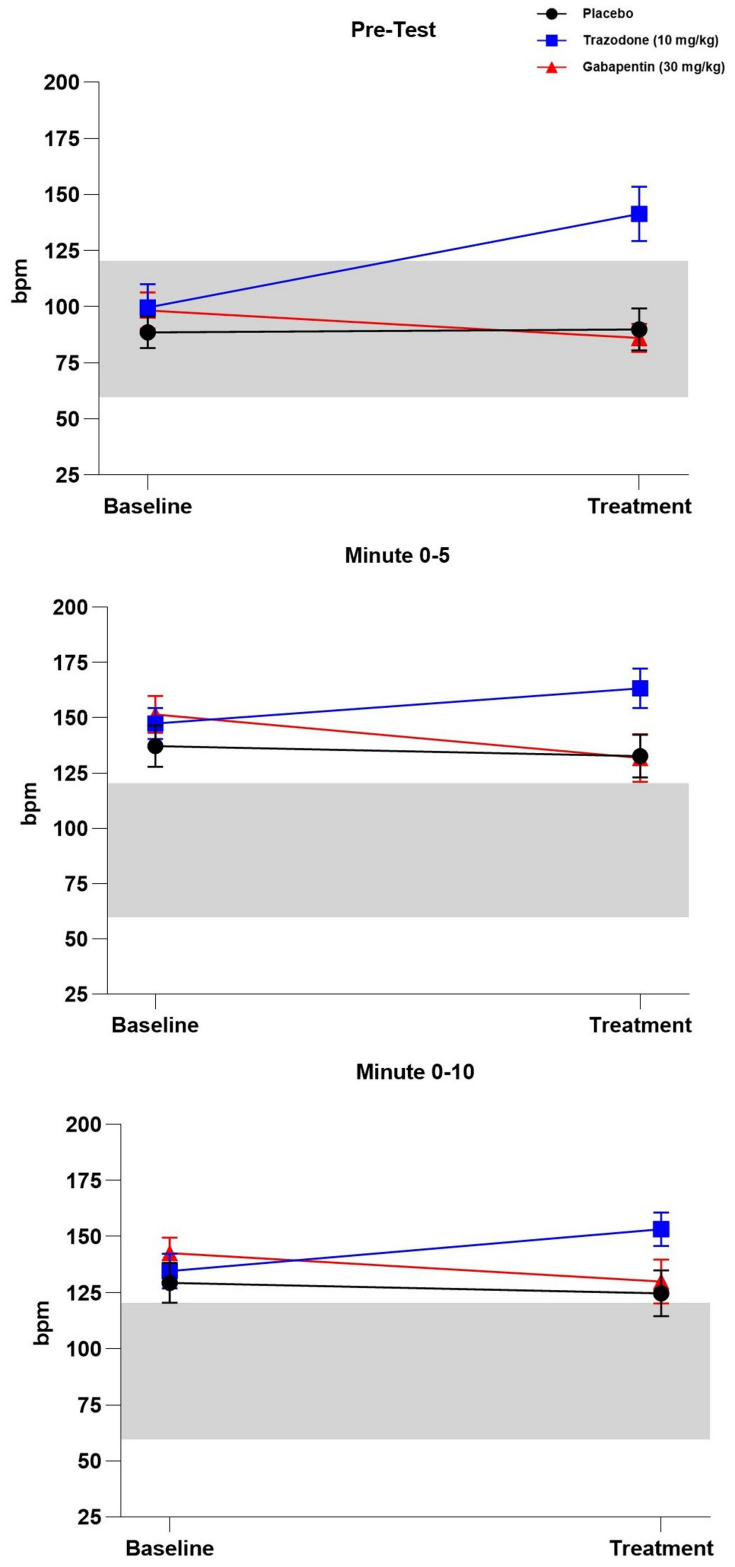
Mean ± SEM heart rate (bpm) by treatment type and study phase, measured pre-test (top panel) and during travel for minutes 0–5 (middle panel) and minutes 0–10 (bottom panel). Adjusted mean heart rate during the treatment phase was significantly higher in the trazodone group than in the gabapentin group during pre-travel, travel minutes 0–5, and travel minutes 0–10 (*p* ≤ 0.02). Compared to the control group, the trazodone treatment group also showed significantly higher heart rates pre-travel (*p* = 0.004) and higher heart rates during travel at minutes 0–5 (*p* = 0.08), 6–10 (*p* = 0.08), and 0–10 (*p* = 0.054). The shaded area represents a normal resting heart range of 60–120 bpm.

Adjusted mean heart rate during the treatment phase was significantly higher in the trazodone group than in the gabapentin group during pre-travel, travel minutes 0–5, and travel minutes 0–10 by 48 bpm (95%CI 17–79), 32 bpm (95%CI 9–56), and 26 bpm (95%CI 4–48), respectively (*p* ≤ 0.02, Figure 6). Compared to the control group, the trazodone treatment group also had a significantly higher adjusted mean heart rate pre-travel by 44 bpm (95%CI 13–74) (*p* = 0.004) and a higher adjusted mean heart rate during travel at minutes 0–5 (*p* = 0.08), 6–10 (*p* = 0.08), and 0–10 (*p* = 0.054) by 21 bpm (95%CI −2 to 43), 21 bpm (95%CI −2 to 43), and 21 bpm (95%CI −0.3 to 42), respectively.

### 3.5 Behavior measures

#### 3.5.1 Global anxiety scores

**Figure 7:**
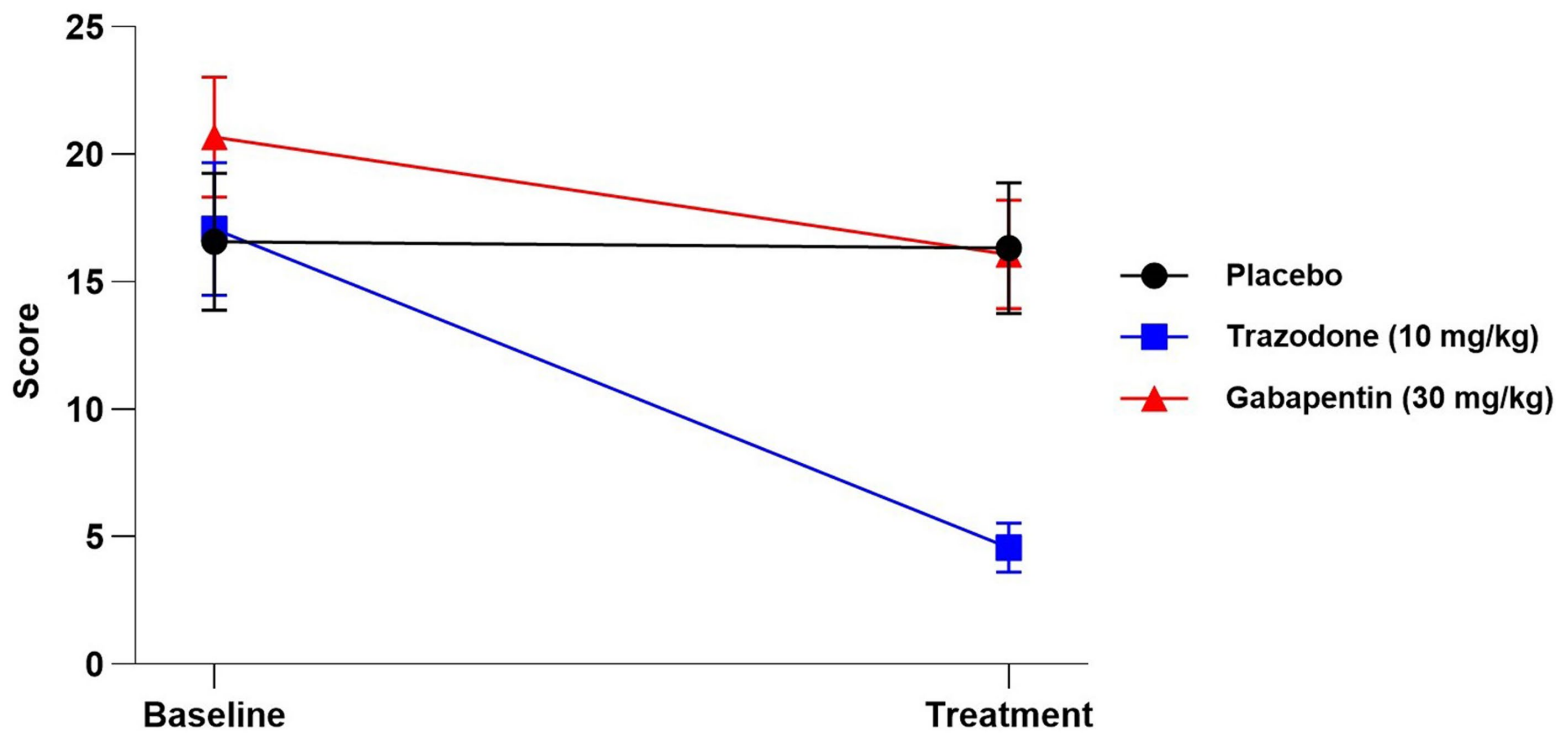
Mean ± SEM global anxiety scores during travel by treatment type and study phase. Adjusted mean global anxiety score was significantly lower in the trazodone-treated group than in the gabapentin- and control-treated groups (*p* < 0.001).

The adjusted mean global anxiety score was significantly lower in the trazodone-treated group than in the gabapentin- and control-treated groups by 9 (95%CI 5–14), and 12 (7–17), respectively (*p* < 0.001; Figure 7). There was also a significant difference in the number of dogs that showed a decrease in global anxiety scores between the groups (*p* = 0.01), with 50% in the control group, 83% in the gabapentin group, and 100% in the trazodone group showing reduction in global anxiety score.

The global anxiety score was calculated as the sum of scores from lip licking, vocalization, salivation, panting, yawning, restlessness/turning and escape attempts (Table 6). At baseline, all dogs exhibited lip licking and 23/36 (64%) exhibited yawning (analyzed independently below). In contrast, vocalization, panting, salivating and escape behaviors were observed in one-third or less of all dogs at baseline. While not initially designated as initial measures, restless behavior and yawning also occurred with sufficient frequency to warrant *post-hoc* analysis. Together with the lip licking score, reductions in yawning, salivation, panting and restlessness scores contributed most to the significant reduction in global anxiety scores observed with trazodone treatment compared to gabapentin and control.

#### 3.5.2 Lip licking

**Figure 8:**
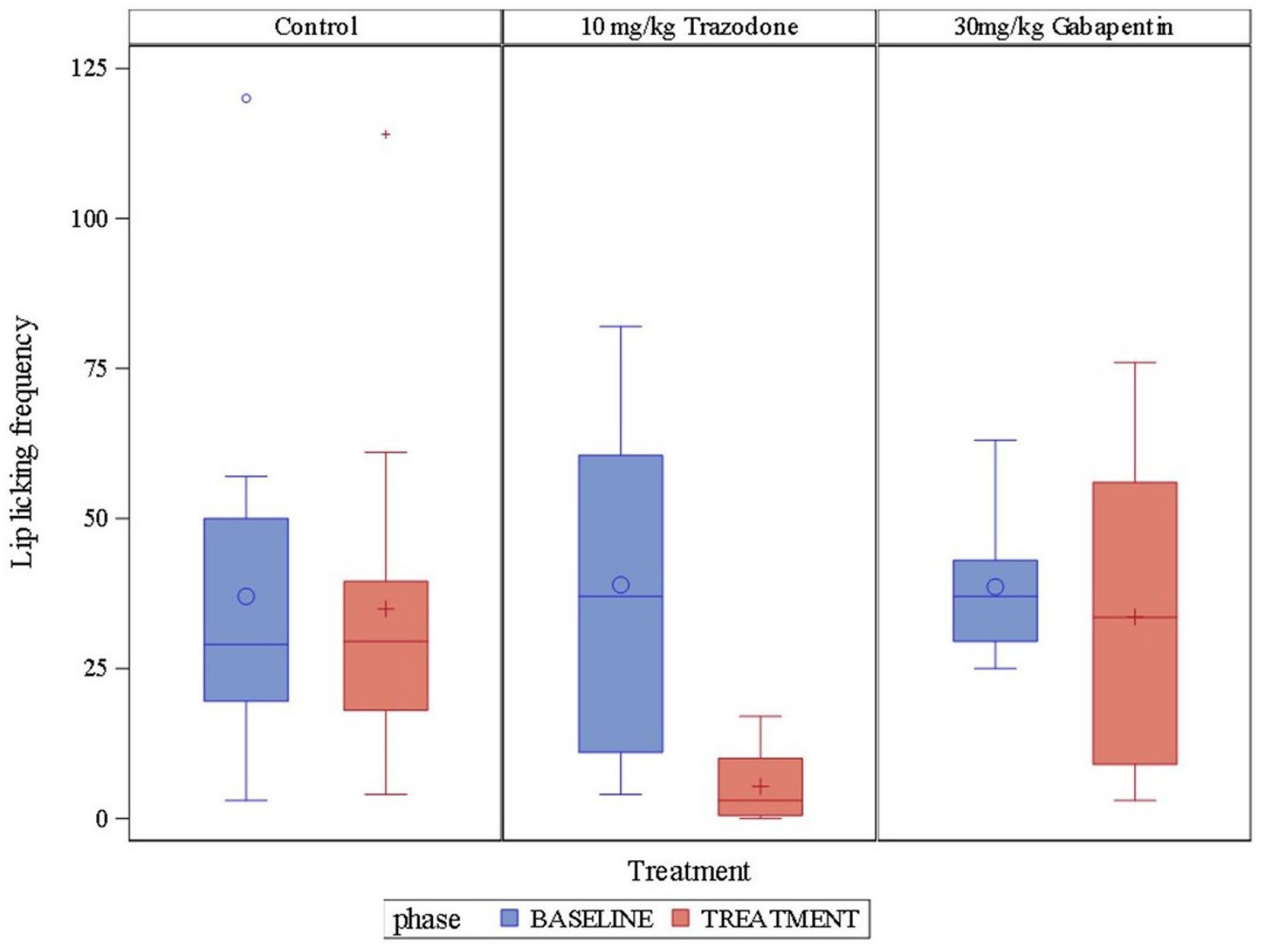
Box plots of lip licking frequency during travel by treatment type and study phase. Adjusted lip licking frequencies and converted lip licking scores were significantly lower in the trazodone-treated group compared to both gabapentin and control (*p* ≤ 0.002).

Adjusted lip licking frequencies and converted lip licking scores were significantly lower in the trazodone-treated group (median frequency = 3.0) than in the gabapentin (median frequency = 33.5) and control (median frequency = 29.5) groups (*p* ≤ 0.002, Figure 8). There was also a significant difference in the number of dogs that showed a decrease in lip licking frequency between groups (*p* = 0.02), with 50% in the gabapentin group, 58% in the control group, and 100% in the trazodone group.

#### 3.5.3 Yawning

Adjusting for baseline phase, there was a significant decrease in the number of dogs that yawned during travel in the treatment phase in the trazodone group (6 baseline, 2 treatment) compared to both control (7 baseline, 8 treatment) (*p* = 0.01) and the gabapentin (10 baseline, 10 treatment) (*p* = 0.002) groups (Table 6).

#### 3.5.4 Restlessness

Adjusted restless behavior scores were significantly lower in the trazodone-treated group with an adjusted mean 1.9 (95%CI 0.2–3.6) lower than the gabapentin group (*p* = 0.03) and 2.3 (95%CI 0.7–4.0) lower than the control group (*p* = 0.005, Table 6).

#### 3.5.5 Changes in body position

Trazodone treatment resulted in significantly lower standing duration compared to gabapentin (45 s decrease in median) (*p* = 0.03) and a significantly greater decrease from baseline in standing duration (127 s increase in median decrease) (*p* = 0.02), as well as a non-significant reduction in standing duration compared to the control group (77 s decrease in median) (*p* = 0.09).

#### 3.5.6 Urination, defecation, and emesis events

Adjusting for baseline phase, there was a significant decrease in the number of dogs that urinated during travel in the treatment phase in the trazodone group compared to the control group and the gabapentin group. In the treatment phase, there were no dogs (0%) that urinated in the trazodone group (compared to five dogs (42%) at baseline), while urination decreased from baseline in the control group from six dogs (50%) to five (42%), and increased in the gabapentin group from five dogs (42%) to eight (75%). The gabapentin group also showed an increase in the emesis during the treatment phase, all of which occurred during travel, to four of 12 (25%) dogs from none (0%) at baseline, whereas the trazodone group decreased to none (0%) during the treatment phase from one (8%) at baseline, and the control group remained the same with one (8%) dog at both treatment and baseline. The frequency of dogs defecating during travel was low (3 events during baseline, 2 events during treatment) and did not result in any treatment-related trends.

### 3.6 Health observations

No adverse health events attributable to treatment were observed during the study. Vomiting associated events occurred during travel assessments and based on their timing and context, were considered likely to reflect travel-related responses rather than a direct treatment-related effect. No signs of sedation were reported in any of the dogs from the time of dosing through to the completion of the travel tests. No animals met the predefined travel-related humane endpoint criteria during the treatment phase of the study.

## 4 Discussion

The laboratory model of road travel-induced anxiety provides a standardized methodology for evaluating the effects of therapeutic interventions by assessing both behavioral and physiological stress measures. Increased cortisol levels have been demonstrated to be a consistent and reliable physiological measure of stress in response to travel, and attenuation of the increases can be used for the assessment of therapeutic response, with improvements seen following treatment with clomipramine (4), CBD (3, 6), and dexmedetomidine oromucosal gel (10). Flint et al. (31) further demonstrated that cortisol elevation is associated with negative valence emotions compared to positive ones, regardless of arousal level, supporting its use as an index of negative affect in this model (31). Thus, the primary outcome measure used to characterize the effect of treatment in the current travel anxiety model was a reduction of post-travel serum cortisol levels. Trazodone, but not gabapentin, significantly decreased post-travel serum cortisol demonstrating anxiolytic effects on this physiological measure of stress.

In the current study, serum cortisol sampling was performed from 8 to 10 min following transport, which was selected based on previous studies where significant increases in salivary, plasma, or serum cortisol were demonstrated within 10 min following the end of the car ride (2–4, 6, 10, 32). In those studies, serum cortisol levels returned to baseline within 30–60 min of the end of travel even for travel lasting up to 2 h (2, 32). Increased levels of cortisol at 10 min following exposure to stressors reflect an adaptive stress response, whereas elevations at 40 min reflect unsuccessful coping or a maladaptive response (33); therefore, the serum cortisol levels seen within 10 min of the end of travel in this study are indicative of an adaptive response.

While some studies have assessed cortisol levels in saliva to reduce blood collection stress (2, 5), serum cortisol (3, 6) was utilized in the current study because of the familiarity of both the animals and technical staff with blood collection, which allowed for successful, low-stress, blood draws of the required volume. As cortisol levels are analyzed and received from the diagnostic laboratory the next day, this also enabled rapid subject selection and balanced allocation to treatment groups. Furthermore, given the type and scope of the research, blood samples for additional analyses, such as for pharmacokinetics evaluation, are often simultaneously collected at the time of cortisol sampling.

Behavioral validity is a critical component of welfare-relevant anxiety models, as reliably observable behaviors are reported to be the standard for assessing welfare (34). However, behavioral responses are highly variable between stimuli, stressors, environments and contexts, and additionally vary due to individual differences and prior experience. Therefore, to be included as measures, the behaviors must be reliably observable, measurable, and consistent across repeated exposures (31). Lip licking is the most consistently and frequently observed sign of stress and frustration across all travel anxiety studies, (3–6, 9–11, 32), and has been demonstrated to be alleviated by anxiolytic therapeutic interventions (3, 4, 6, 9–11). Beyond its frequency and consistency, lip licking has been associated with negative affective states in dogs in response to aversive stimuli and emotional conflict and demonstrating sensitivity to anxiolytic intervention (3, 31). In the current study, trazodone significantly reduced lip licking frequency and lip licking scores compared to both gabapentin and the negative control. Other behavioral measures, including panting, yawning, vocalization, and restlessness, are more variable, less consistently observed, often have a higher rate of low occurrences, or are less likely to be maintained across repeated travel (3, 5, 6, 31). Lip licking is therefore the primary behavioral measure, that we assessed independently in terms of both frequency and as a converted score (magnitude). A global score was then calculated from the total of all lip licking score and the other behavior measures of yawning, panting, vocalization, salivating, restlessness, and escape (Table 5). The only behavioral measures, other than lip licking, reported to be significantly reduced by therapeutic interventions in travel studies are panting, yawning and whining (3, 4, 9, 40). In this study, fewer than 30% of dogs showed panting or vocalization at baseline. However, over 60% of dogs exhibited yawning at baseline, which was significantly reduced with trazodone treatment relative to both gabapentin and control. In addition, restless behavior scores were significantly lower in trazodone-treated dogs than in either comparison group. We also observed a near significant reduction in standing duration with trazodone compared to control. Reduced restlessness and decreased standing duration are consistent with a reduction in arousal-related vigilance and anxiety, as dogs experiencing negative affective states typically exhibit increased motor activity and difficulty settling in aversive contexts (31).

Observation of excreta events during travel is primarily used for animal welfare monitoring; however, in the current study, there was a significant decrease in the number of dogs that urinated in the trazodone-treated group. This finding further supports the reduction in anxiety under trazodone treatment, as increased urination is an observed physiological response to stress in dogs (34). As trazodone does not have anticholinergic effects (18), the decrease in urination is unlikely to be related to urine retention, and no urine retention was observed in the present study. As the increase in emesis events from baseline in the gabapentin-treated group during the treatment phase was observed exclusively during travel, with no events between dosing and travel, this suggests that these events were more likely related to individual susceptibility to motion during transport rather than a direct pharmacological effect of gabapentin. Emesis is not a reported side effect of gabapentin in dogs (25), nor was there any vomiting in the 18 dogs in the thunderstorm study at 25–30 mg/kg (29), and only one reported event at 15 min post dosing in the 22 dogs in the veterinary visit study at 50 mg/kg (16). These findings likely reflect a lack of efficacy of gabapentin in mitigating travel-associated gastrointestinal responses under the conditions tested, rather than an adverse treatment-related effect.

The doses of trazodone and gabapentin tested in this study were selected based on previous research assessing their individual effects on situational fear and stress, including veterinary visits, hospitalization, and noise fears (14, 16, 22, 26, 29) and on literature describing their use in clinical practice (13, 15). In addition, we selected 2 h following administration to conduct the transport test to correspond with the peak effects (Tmax) of each compound. Clinical studies have demonstrated anxiolytic effects of gabapentin occurring 90 min to 2 h after administration (16, 29), while pharmacokinetic studies indicate that gabapentin has a Tmax of 1.1–1.5 h, an onset of action reported between 30 and 90 min and a duration of approximately 7–8 h (27, 35, 36). For trazodone, calming effects are reported 1–2 h after administration (14, 37), while pharmacokinetic studies indicate a Tmax of approximately 2.5 h (38); however, individual variability is high, with a recent study of six dogs reporting a range for Tmax of 445 (±271) minutes, and ranging from 30 min to 10 h for dogs receiving 8 mg/kg orally (39).

This study has several limitations. The sample size was based on prior canine travel and situational studies in which cortisol and behavioral signs were significantly improved (6, 10, 29, 40) and informed in part by *a priori* power analysis with cortisol as the primary endpoint, in an unpublished internal study using the same road travel model. While this approach supported adequate power for detecting changes in cortisol, the sample size may have been insufficient to detect smaller effects in secondary and exploratory endpoints. The study employed an acute, single-dose design in a controlled laboratory model, and therefore does not address the effects of repeated dosing or longer-term management of travel-related anxiety. While treatment groups were balanced based on baseline cortisol response and recent travel exposure, other sources of variability, including sex, age, and individual differences in stress responsiveness, were not included as covariates in the statistical models. As such, the potential influence of these factors on treatment response cannot be fully excluded. In addition, although heart rate provided a useful physiological measure of arousal during transport, intermittent loss of sensor functionality resulted in missing data at some time points. Missing heart rate data were primarily due to loss of sensor contact due to changes in body position and animal movement into the vehicle and during transport, which is consistent with known limitations of chest-strap monitoring in dogs under dynamic conditions. While Polar heart rate monitoring has demonstrated accuracy for dogs that are stationary or exhibiting typical movement, intermittent data loss due to interruption of electrode contact and signal conduction is a reported limitation of these devices during periods of increased activity or irregular movement, such as during road travel on unpaved surfaces or when dogs shift, brace, or make corrective movements to maintain stability in response to vehicle motion (41, 42). The distribution of missing data across treatment groups was limited and did not show a clear pattern suggestive of systematic bias. As analyses were performed using available observations for each endpoint and timepoint, the impact of missing data on statistical inference is expected to be minimal, particularly during travel in which no more than one dog per group had missing data. Finally, the use of a standardized laboratory Beagle model enhances experimental control but may limit direct extrapolation to the broader population of client-owned dogs and diverse real-world travel environments.

While the administration of 10 mg/kg trazodone significantly reduced post-travel serum cortisol, global anxiety scores, lip licking frequencies, lip licking scores, restlessness scores, cortisol responses, urination events, and the number of dogs with decreased global anxiety, lip licking and yawning, alleviation of fear and anxiety during travel would also be expected to lead to a reduction in heart rate. In fact, using the same study design, a significant decrease in pre- and during-travel heart rates has previously been demonstrated with dexmedetomidine oromucosal gel treatment compared to control (10). However, in this study both pre- and during-travel heart rate were found to be increased under treatment with trazodone. This elevation is consistent with other studies, in which increased heart rate was found to be strongly correlated with higher plasma levels of trazodone (39), at 2 h after a single dose of 9–12 mg/kg (14) and at a dose of 6 mg/kg three times a day over 24 h (43). At higher doses, trazodone may exhibit significant alpha-adrenergic blocking, primarily antagonizing alpha-1 adrenoreceptors. Blocking alpha-1 adrenergic receptors results in vasodilation and hypotensive effects which may lead to a compensatory or reflex tachycardia (43, 44). However, trazodone may also moderately antagonize alpha-2 receptors which would increase the release of norepinephrine, stimulate heart rate, and increase blood pressure. As neither trazodone levels nor blood pressure were collected, their relationship to heart rate has not been assessed and proof of mechanism has not been established. Another explanation might be a serotonin-mediated effect on heart rate (43). Inter-individual variability in pharmacokinetic and pharmacodynamic responses to trazodone could also contribute to differences in heart rate. Assessment of heart rate variability might also provide insight into autonomic balance, helping to distinguish between sympathetic activation associated with stress and pharmacologically mediated changes in heart rate. In addition, future studies assessing heart rate in a trazodone-only condition without a concurrent stressor, could further help to differentiate direct pharmacological effects on heart rate from a physiologic response, while investigations with lower trazodone doses could help determine if stress reduction can be achieved without an accompanying increase in heart rate. Therefore, while the increased heart rate may be attributable to pharmacologic and autonomic effects rather than stress related activation, the exact mechanism, clinical relevance and optimal dose cannot be confirmed based on the available data.

There were no significant effects on any of the behavioral or physiological measures with gabapentin at the dose and timing evaluated in this study (a single dose of 30 mg/kg administered 2 h prior to travel). The absence of an observed effect in this study should therefore be interpreted within the context of the specific regimen evaluated. It is possible that alternative dosing strategies, including higher doses [e.g., 50 mg/kg as reported by Stollar et al. (16)] or different timing relative to the stressor may warrant further investigation.

## 5 Conclusion

The results of this placebo-controlled study demonstrate that the described model provides a standardized design and methodology for evaluating the effectiveness of therapeutics on the behavioral and physiological signs of fear, anxiety, and stress during road travel. Treatment with 10 mg/kg trazodone, administered 2 h prior to travel, resulted in significant reductions in cortisol response and multiple observable behavioral signs of stress, including lip licking, global anxiety score, urination, yawning and restlessness compared to placebo and gabapentin. These findings strongly support the efficacy of a single dose of trazodone in alleviating travel anxiety and stress. While an elevation in heart rate was observed, available research suggests this is likely to be a pharmacologic/autonomic side effect, rather than sympathetic stress activation. There were no measurable effects of gabapentin at 30 mg/kg under the conditions evaluated in this study.

## Data Availability

The raw data supporting the conclusions of this article will be made available by the authors, without undue reservation.
